# Effect of wing length on the prevalence of trypanosomes in *Glossina morsitans morsitans* in eastern Zambia

**DOI:** 10.1186/s13071-021-04907-y

**Published:** 2021-08-18

**Authors:** Cornelius Mweempwa, Kalinga Chilongo, Kyoko Hayashida, Boniface Namangala

**Affiliations:** 1 Department of Veterinary Tropical Diseases, Faculty of Veterinary Science, University of Pretoria, Pretoria, South Africa; 2 Department of Veterinary Services, Tsetse and Trypanosomiasis Control Section, Ministry of Fisheries and Livestock, Lusaka, Zambia; 3 Division of Collaboration and Education, International Institute for Zoonosis Control, Hokkaido University, Sapporo, Hokkaido Japan; 4 Department of Paraclinical Studies, School of Veterinary Medicine, University of Zambia, Lusaka, Zambia

**Keywords:** Wing length, Prevalence of trypanosomes, *Glossina morsitans morsitans*, Tsetse sampling methods

## Abstract

**Background:**

Tsetse flies (Diptera: *Glossinidae*) transmit trypanosomiasis (sleeping sickness in humans and nagana in livestock). Several studies have indicated that age, sex, site of capture, starvation and microbiome symbionts, among others, are important factors that influence trypanosome infection in tsetse flies. However, reasons for a higher infection rate in females than in males still largely remain unknown. Considering that tsetse species and sexes of larger body size are the most mobile and the most available to stationary baits, it was hypothesized in this study that the higher trypanosome prevalence in female than in male tsetse flies was a consequence of females being larger than males.

**Methods:**

Black screen fly rounds and Epsilon traps were used to collect tsetse flies in eastern Zambia. Measurement of wing vein length and examination for presence of trypanosomes in the flies were carried out by microscopy. Principal component method was carried out to assess the potential of wing vein length as a predictor variable. The multilevel binary logistic regression method was applied on whole data, one-method data and one-sex data sets to evaluate the hypothesis.

**Results:**

Data derived from a total of 2195 *Glossina morsitans morsitans* were evaluated (1491 males and 704 females). The wing length variable contributed the highest variance percentage (39.2%) to the first principal component. The variable showed significant influence on prevalence of trypanosomes when the analysis was applied on the whole data set, with the log odds for the prevalence of trypanosomes significantly increasing by 0.1 (*P * =  0.032), per unit increase in wing length. Females had higher trypanosome prevalence rates than males, though not always significant. Furthermore, moving from females to males, wing length significantly reduced by 0.2 (*P * <  0.0001).

**Conclusions:**

We conclude that wing length is an important predictor variable for trypanosome prevalence in *Glossina morsitans morsitans* and could partially explain the higher prevalence of trypanosomes in females than in males. However, reasonably representative population data are required for analysis—a serious challenge with the current tsetse sampling methods. Thus, analysis combining data from mobile and stationary methods that include both sexes' data could be useful to verify this hypothesis.

**Graphical abstract:**

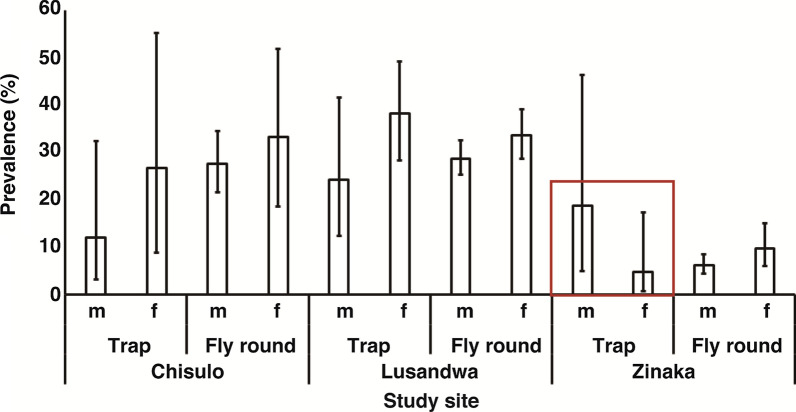

**Supplementary Information:**

The online version contains supplementary material available at 10.1186/s13071-021-04907-y.

## Background

Tsetse flies (Diptera: *Glossinidae*) transmit trypanosomiasis, a neglected tropical disease (NTD) [1]. Sampling of tsetse flies, and examination for trypanosomes in them, is a prerequisite for studies on demography, biometrics, genetics, epidemiology, diagnostics and therapeutics, in relation to management of trypanosomiasis. Sampling tsetse flies involves the use of stationary and/or mobile techniques. These techniques are known to display inherent biases toward certain sections of populations, e.g. toward particular species, sex, age group and pregnancy stage [2, 3]. For example, stationary traps are known to sample more *Glossina pallidipes* than *Glossina morsitans morsitans*, more females than males and more old than young flies [2, 3]. On the other hand, mobile methods (such as “fly rounds”) are known to sample more *G. m. morsitans* than *G. pallidipes*, more males than females and more young than old flies [2, 3]. Stationary methods (trap devices) are known to sample more of the larger than the small flies [4]. In a study by Hargrove [2], age structures determined from samples of tsetse flies caught using different methods differed significantly in six out of ten pair comparisons in *G. m. morsitans* and in all ten pair comparisons in *G. pallidipes*, and the differences were attributed to increased flight activity with age. Some studies have been carried out to identify factors that influence rates of trypanosome infection in tsetse flies and these factors include age [5], sex and site of capture [6], starvation [7] and microbiome symbionts [8, 9] among others.

As species and sexes of tsetse flies of larger body size are the most mobile [10] and are also the most available to stationary baits [11], it was hypothesized in this study that the higher rate of trypanosome prevalence in female than in male tsetse flies described in *G. pallidipes* [12], *Glossina palpalis palpalis*, *Glossina tachinoides* and *Glossina morsitans submorstans* [6] was a consequence of females being larger than males on average, with the smallest females being smaller than the largest males [13]. However, in laboratory-bred *G. m. morsitans*, infection rates by trypanosomes of the subgenus *Trypanozoon* in salivary glands were shown to be higher in males than in females [14, 15]. Considering that body size was shown to have an influence on mobility and females tend to be larger than males, this study was conducted to investigate whether body size (measured as wing vein length) had an influence on prevalence of trypanosomes in tsetse flies.

## Methods

### Study area and sampling of tsetse flies

The data were collected in eastern Zambia during a study on the status of tsetse populations and epidemiology of livestock trypanosomiasis in areas of varying degrees of habitat fragmentation [16]. The description of the study area, methods used to sample tsetse populations and laboratory examinations of tsetse flies were as provided in previous studies in the area [4, 17, 18].

In brief, sampling of tsetse flies was carried out monthly for 1 year from four sites of varying levels of habitat fragmentation [18]. The degree of fragmentation increased in the order Lusandwa, Zinaka, Chisulo and Kasamanda study sites [17, 18]. The shortest and longest distances apart between study sites were 10 and 30 km, respectively. Tsetse density increased as degree of fragmentation reduced [17]. Black screen fly rounds [19] and Epsilon traps [20] were used to sample tsetse populations. Collected tsetse flies were subjected to (i) sorting by sex, (ii) examination for trypanosome infection by microscopy [21], (iii) measurement of wing vein length [22], (iv) dissection of females for ovarian aging [23] and (v) assessment of wing fray for aging purposes in males [24].

### Data analysis

Although two tsetse species (*G. m. morsitans* and *G. pallidipes*) were collected in the study area [18], only *G. m. morsitans* data, from both sampling methods (fly rounds and Epsilon traps), were subjected to the analyses. Very few *G. pallidipes* were collected and not at all the sites. Few flies (both species) were caught in the Kasamanda site, and consequently the data were excluded from the study. In the analyses, data from the three sites considered (Chisulo, Lusandwa and Zinaka) were pooled.

In analysis of the influence of wing length on prevalence of trypanosomes in *G. m. morsitans*, the data used were from all flies examined for presence of trypanosomes that also had their right wing length measured (the measurement of the left wing was used where the right wing measurement was missing). Considering the type of data usually available under field conditions, three types of data sets that differed in inclusiveness of variables of interest were analyzed. First were wing length data from both sampling methods (i.e. fly rounds and traps—pooled) and from both sexes, here referred to as the “whole data set.” Second were wing length data from only one method (i.e. either fly round or trap) but with both sexes' data included, here referred to as “one-method data set,” and lastly, data from one sex (i.e. either males or females) but both methods' or one method's data analyzed, herein referred to as “one-sex data set.”

Because the data sets were clustered in study sites, multilevel binary logistic regression was applied taking study site as a random variable. Permutation logistic regression models on prevalence of trypanosomes were run for the different data sets. The predictor variables used included method, season, sex and wing vein length (herein referred to as wing length). The variable wing length was standardized prior to analysis. The Levene’s test for equality of variance was applied on wing length data between males and females for respective sites before application of the Student’s and Welch’s t test (an adaptation of the former) to compare the means. Additional analyses were carried out to gain more insight and confidence into the results. The likelihood ratio test was carried out to compare models and determine the best fit. For each data set, the less than full models were each compared with the full model.

The variable inflation factor (VIF) was calculated to check for multicollinearity among the various model variables. Predictor variables with VIF values > 5 were considered to have multicollinearity issues, i.e. they made the model less able to explain the relationship between the response and predictor variables.

The principal component method, multiple factor analysis [25] in particular, was carried out to determine how much variance each predictor variable contributed to the first principal component as a way to tell whether a variable was a potential predictor. Variables that contributed most variance to the first principal component were considered potential predictors [26]. The sample size used in each model was checked for sufficiency for use in logistic regression analysis using methods of Bujang et al. [27] and Peduzzi et al. [28]. The R statistical software [29] was used in the analysis.

## Results

### Numbers of flies collected and infected

A total of 2375 *G. m. morsitans* had their wing vein length measured, and 2464 were dissected for trypanosome infection examination, of which 2195 (1491 males and 704 females) were used in multilevel binary regression and principal component analyses (Table 1).

**Table 1:** Status of trypanosomes presence in *Glossina morsitans morsitans* in eastern Zambia

Study site	Cold season		Hot season		Rainy season	
	Negative	Positive	Negative	Positive	Negative	Positive
Chisulo	73	23	13	8	113	42
Lusandwa	243	121	71	30	427	180
Zinaka	302	18	103	13	385	30
Total	618	162	187	51	925	252

### Wing length and prevalence of trypanosome infection

**Fig. 1:**
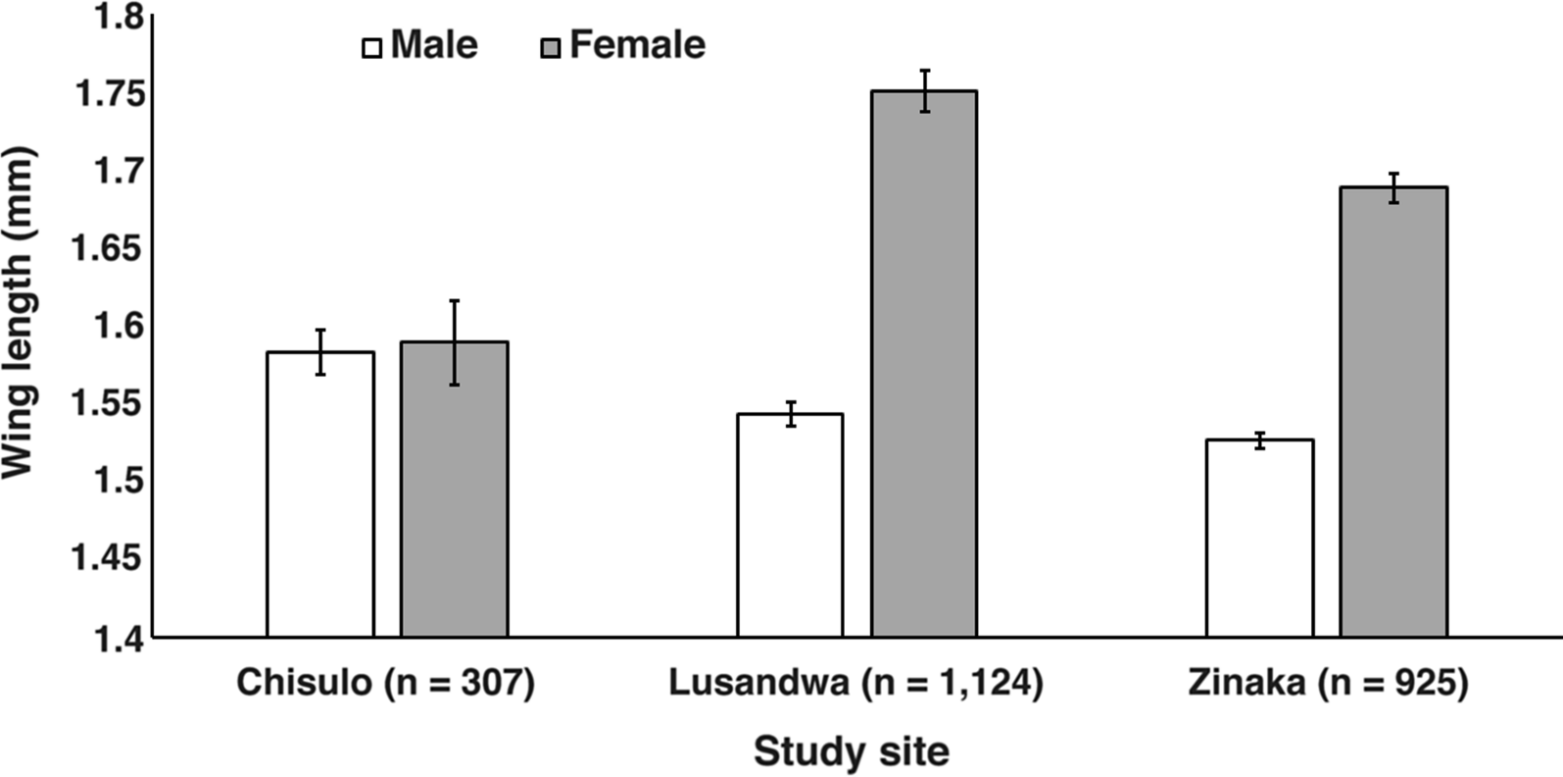
Mean wing length in male and female *Glossina morsitans morsitans* in eastern Zambia

**Fig. 2:**
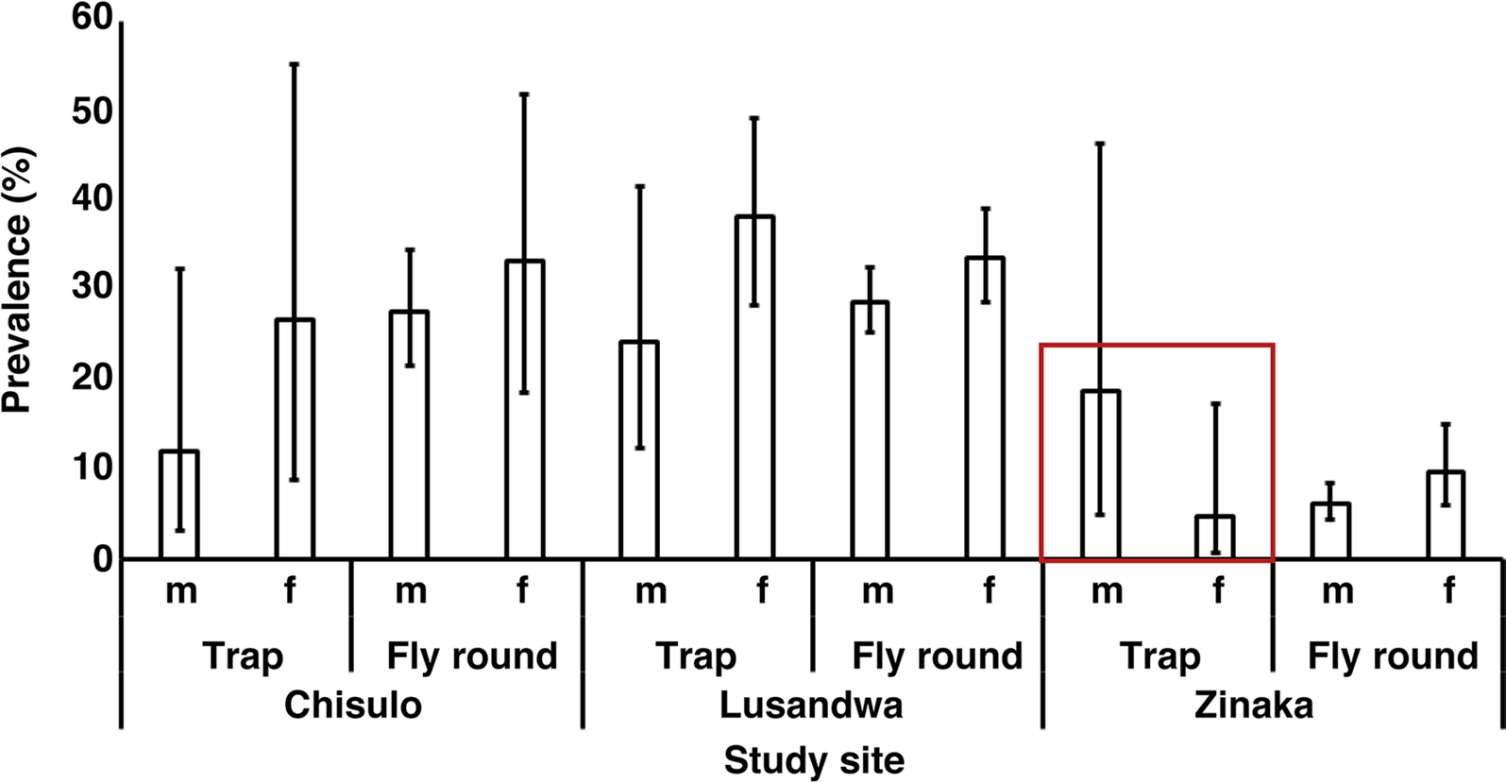
Prevalence of trypanosomes in *Glossina morsitans morsitans* in eastern Zambia

As Fig. 1 shows, the Lusandwa site recorded the longest mean wing length in the females (1.75 mm) whilst Chisulo site had the longest mean wing length in the male flies (1.58 mm). Unequal variance in wing lengths between males and females was observed on Lusandwa and Zinaka sites samples, *F*_(11,122)_  =  38.0 and *F*_(1923)_  =  7.8, respectively, *P * <  0.0001. The Chisulo site samples had equal wing length variance between males and females, *F*_(1305)_  =  0.1, *P * =  0.7801. The Welch’s *t* test results showed that the mean wing length was significantly longer in females than in males at Lusandwa and Zinaka, *t*_(768.3)_  =  25.8 and *t*_(419.9)_  =  29.7, respectively (*P * <  0.0001), with degrees of freedom shown in parentheses. No significant difference in mean wing length between females and males at Chisulo site was observed after applying the Student’s *t* test, *t*_(305)_  =  0.4 (*P * =  0.6989). Regarding prevalence of trypanosomes, except for trap results in Zinaka (red box), female flies had higher prevalence than male flies at all study sites and sampling methods (Fig. 2). The difference in prevalence was significant between those of male and female flies caught from fly rounds in Zinaka and those of counterpart sexes at Chisulo and Lusandwa as evidenced by the non-overlapping 95% confidence intervals.

### Multilevel binary logistic regression analysis

The multilevel binary logistic regression results showed that the wing length was an important predictor variable of the prevalence of trypanosomes in *G. m. morsitans*. However, this was only true in some permutation models (3/4) of only the whole data set, which did not have sex as one of the predictor variables. In models that had both the wing length and sex as predictor variables (4), both variables did not show significant influence on the prevalence of trypanosomes (*P*  >  0.360 and 0.078, respectively). In the permutation model where the wing length variable showed the strongest influence on prevalence of trypanosomes, i.e. the one that had the method, season and wing length as predictor variables, model 1c (Table 2), for per unit increase in wing length, the log odds for the prevalence of trypanosomes significantly increased by 0.1 (*P * =  0.032). The wing length variable of the model with method and wing length as the only predictor variables showed the weakest but significant influence on prevalence of trypanosome, per unit increase in wing length; the log odds significantly increased by 0.1 (*P * =  0.044—model results not shown).

**Table 2:** Multilevel binary logistic regression permutation models with special reference to the wing length and the sex variables

Logistic model	Predictor	Coefficient	*P *value	AIC
1.0 whole data set				
Full model (*n * = 2195)	Trap method	− 0.1	0.562	2100.9
	Hot season	0.2	0.317	
	Rainy season	− 0.1	0.673	
	Male sex	− 0.2	0.090	
	Wing length	0.1	0.370	
Model without wing length predictor	Trap method	0.0	0.799	2099.7
	Hot season	0.2	0.348	
	Rainy season	0.0	0.701	
	Male sex	− 0.3	0.010*	
Model without sex predictor	Trap method	− 0.1	0.526	2101.8
	Hot season	0.2	0.311	
	Rainy season	− 0.1	0.631	
	Wing length	0.1	0.032*	
2.0 one-method data set: fly round				
Full model (*n * = 1971)	Hot season	0.2	0.273	1863.7
	Rainy season	− 0.1	0.478	
	Male sex	− 0.2	0.146	
	Wing length	0.1	0.545	
Model without wing length predictor	Hot season	0.2	0.296	1862.1
	Rainy season	− 0.1	0.467	
	Male sex	− 0.3	0.024*	
Model without sex variable	Hot season	0.2	0.256	1863.8
	Rainy season	− 0.1	0.482	
	Wing length	0.1	0.069	

*P  <  0.05 significance level

In permutation models for the one-method data set (4), i.e. fly round data set, per unit increase in wing length, there too were increases in the log odds by 0.1 as shown in a representative model (Table 2, model 1c). However, these increases were not significant (*P * >  0.068). No logistic regression analysis was run on the trap-only data set because the sample size of 224 was lower than the minimum of 250 required for the regression analysis. The wing length variable did not show significant influence on prevalence of trypanosomes in all other permutation models of other data sets that had sufficient sample sizes for analysis.

The sex variable also showed significant influence on prevalence of trypanosomes as exemplified in permutation models without the wing length as one of the predictor variables, built from both whole data set (4/4) and one-method (fly round) data sets (2/2). Representative models are 1b and 2b, respectively (Table 2). The log odds for prevalence of trypanosomes significantly reduced by 0.3 (*P * =  0.010) on moving from females to males in the model (1b) with the weakest but significant influence on prevalence of trypanosomes for the whole data set and by 0.3 (*P * =  0.024) in the model (2b) with the weakest influence on prevalence of trypanosomes for the one-method (fly round) data set. In these models without wing length as one of the predictor variables, females had significantly higher trypanosome prevalence rates than males as noted from the negative log odds values of the sex variable (Table 2). Females did not have significantly higher trypanosome prevalence rates than males in permutation models (4/4 for whole data set and 2/2 for fly round data set) that included the wing length variable as one of the predictor variables as shown by representative models 1a and 2a, respectively (Table 2).

A linear regression analysis on wing length where the sampling method, season, sex and study site were used as predictor variables for the whole data set showed that, moving from females to males, the wing length significantly reduced by 0.2 (*P * <  0.0001). For fly round-only and trap-only data sets, moving from females to males, the wing length significantly reduced by 0.2 and 0.1, respectively (*P*  <  0.0001).

In analysis of “females-only” data, in all permutation models of method, season, wing length and ovarian category predictor variables, only the ovarian age category variable showed significant influence on prevalence of trypanosomes (*P * =  0.030). No variable showed significant influence on prevalence in all permutation models when the “males-only” data set was analyzed using the same predictor variables as for the “females-only” data set (ovarian category variable was replaced by the wing fray variable in males-only data analysis).

Results of tests carried out to obtain further information in connection with observed regression analysis were supportive of the observations and are presented below.

### Likelihood ratio test

Results of the likelihood ratio test carried out on permutation models for the whole data set showed that the sex and wing length variables together significantly improved the fit on the data set (*P * =  0.024) from the AIC  =  2104.4 for the model without the two variables to the AIC  =  2100.9 for the model with the two variables—the full model (Table 3). However, this was not the case on similar tests carried out on one method-only (fly round-only) data set. For the one-sex data set, i.e. females-only and males-only, no significant improvements in model fit of the data were observed by including the wing length as one of the predictor variables, *P * =  0.915 and 0.152, respectively (Table 3).

**Table 3:** Likelihood ratio test results between the full and the less-than-full models for each data

Data set	Test no.	Models	AIC	Pr(> Chisq)
Whole data (*n * = 2195)		Full model	2100.9	
	1	No sex and wing length variables model	2104.4	0.024*
	2	No wing length variable model	2099.7	0.371
	3	No sex variable model	2101.8	0.091
Fly round-only data (*n * = 1971		Full model	1863.7	
	1	No sex and wing length variables model	1865.1	0.067
	2	No wing length variable model	1862.1	0.546
	3	No sex variable model	1863.8	0.147
Females-only data set (*n * = 704)		Full model	725.8	
		No wing length variable model	723.8	0.915
Males-only data set (*n * = 1491)		Full model	1334.6	
		No wing length variable model	1334.7	0.152

As for the AIC values of models for the whole data set, the model with the lowest AIC  =  2095.3 was the one where the sex variable was the only predictor variable (showed significant influence on prevalence of trypanosomes, *P * =  0.011), followed by the one where the sex and the wing length variables were the only predictor variables AIC  =  2097.0 (showed no significant influence on prevalence of trypanosomes), *P * =  0.077 and 0.580, respectively. The model with the sex (no wing length) among the predictor variables, (model 1b of Table 2) fitted the data better (AIC  =  2099.7) than the one with wing length (and no sex) among the predictor variables, model 1c (AIC  =  2101.8).

### Variable inflation factor

Results of tests for multicollinearity on full models of all data sets showed that all variable inflation factors (VIF) were < 5 (Additional file 1: Table S1). The wing length variable from the whole data set had the highest VIF of 1.8 and the season variable from the fly round-only data set had the lowest (1.0).

### Principal component analysis

Results of the principal component analysis showed that the wing length variable contributed the highest variance to the first principal component (PC1). However, it was observed that the wing length variable did so only in data sets where, except for the trap-only data set, the variable “method” was among those used in the analysis (Additional file 1: Table S2). These data sets included whole and one sex-only (females-only and males-only) data sets, 39.2, 37.8 and 33.1%, respectively. In analysis of the one-method data sets in which both sexes' data were included, the wing length variable contributed the highest variance (33.2%) to the first PC1 for the trap data set while the sex variable contributed the highest variance [45.8%—almost equal to that for the wing length variable (45.7%)] for the fly round data set. In analyses of the one-sex data sets from one method, the site variable contributed the highest variance to the first PC1 for both methods data sets, except for the males-only fly round data set, where the contribution was similar to that of the wing length variable.

## Discussion

This study showed that wing length was among the important factors that influenced prevalence of trypanosomes in *G. m. morsitans*. Detecting its importance in doing so, however, required that the data set analyzed comprised data from both sampling methods and both sexes, as shown from results of the analysis of the whole data set, model 1c (Table 2). Probably because of the sampling bias inherent in individual methods with respect to wing length [4], analysis of the one-method data set did not show the importance of wing length as an important predictor variable for prevalence of trypanosomes; the influence of the variable on prevalence seemed suppressed in the analysis. Probably, in analysis of methods pooled data, there was compensation for sampling bias shortcomings of one method by the other.

The requirement for analysis of data from the two methods to determine the importance of the wing length variable in influencing the prevalence of trypanosomes was further elucidated by results from the likelihood ratio test (Table 3) and the principal component analysis (Additional file 1: Table S2). The likelihood ratio test results showed the importance of the wing length variable through its contribution to model fit on the whole data set, while the principal component analysis showed it through its contribution to variance in the data pooled from both methods, thereby demonstrating its potential as a predictor variable. The principal component analysis further showed that the potential of the wing length being a predictor variable for the prevalence of trypanosomes was seen even in results of one-sex data sets, as long as the data sets included data from both sampling methods. Furthermore, results of the principal component analysis showed the suppression effect on the wing length as an important predictor variable when one-method data were analyzed. Unlike for the whole data set, the contribution of variance by the wing length variable fell below that of the sex variable when one method-only (fly round-only) data were analyzed. Furthermore, principal component analysis where the analyzed data set was one-sex-only data showed suppressed contribution of variance by the wing length variable (site variable had highest contribution), suggesting that the presence of data for the two sexes was also important to include in the analysis to show that the wing length variable was an important factor for prevalence of trypanosomes in *G. m. morsitans*.

In addition, the lack of multicollinearity or collinearity as noted from the low VIF values (1.0–1.8) was an indication that each variable had a unique contribution and all variables in the models together influenced the prevalence of trypanosomes without worrisome interference from each other.

Because the log odds for prevalence of trypanosomes in *G. m. morsitans* significantly increased by 0.1 (*P * =  0.032) per unit increase in wing length and on moving from females to males, the wing length significantly reduced by 0.2 (*P * <  0.0001), and considering that a *Glossina* sex taxon with larger body size was more mobile than that of the smaller body size [10]—thus increasing the chance of acquiring an infection. Having longer wing length was associated with higher prevalence of trypanosomes, hence the higher prevalence in females than in males, thereby supporting the hypothesis. If at the age when most susceptible to infection, i.e. 24 h post-eclosion, the susceptibility to infection was similar between females and males [30], then at that age susceptibility was less likely to be the cause of higher prevalence in females. Since midgut-only infections formed part of the data analyzed in this study, probably the host preference and the feeding frequency differences between females and males (if any) could be a possible reason for higher prevalence of trypanosomes in females at older age. This study brought forth the wing length variable as not only an important factor that influenced the prevalence of trypanosomes in tsetse but also as an associable reason for higher prevalence in females than in males of *G. m. morsitans*.

The lack of significant difference in mean wing length between male and female flies at Chisulo could be attributed to size-dependent mortality in male flies [31–33] because of high levels of habitat fragmentation [17, 18].

## Conclusions

We conclude that wing length is an important predictor variable for trypanosome prevalence in *G m. morsitans* and could partially explain the higher prevalence of trypanosomes in females than in males. However, reasonably representative population data are required for analysis—a serious challenge with the current tsetse sampling methods. Thus, analysis combining data from mobile and stationary sampling methods that include both sexes' data could be useful to verify this hypothesis.

## Supplementary Information

**Additional file 1: Table S1.** Variable inflation factors for model predictor variables. **Table S2**. Percentage predictor variable contribution of variance to the first three principal components (PCs) for different data sets.

## Data Availability

The datasets during and/or analysed during the current study available from the corresponding author on reasonable request.

## Abbreviations

VIF: Variable inflation factor
PC: Principal component
