# Supplementary material for: Effect of wing length on the prevalence of trypanosomes in Glossina morsitans morsitans in eastern Zambia
Source: Parasit Vectors. 2021 Aug 18;14:409. doi: 10.1186/s13071-021-04907-y (PMC8371877; doi:10.1186/s13071-021-04907-y)
Supplement: Supplementary file 1 — Additional file 1: Table S1. Variable inflation factors for model predictor variables. Table S2. Percentage predictor variable contribution of variance to the first three principal components (PCs) for different data sets. [file 13071_2021_4907_MOESM1_ESM.docx]

**Additional file 1: Table S1.** Variable inflation factors for model predictor variables.

| **Model data** | **Model variables** | **VIF** |
| --- | --- | --- |
| Whole data set (n = 2,195) | Method | 1.3 |
|  | Season | 1.0 |
|  | Sex | 1.5 |
|  | Wing length | 1.8 |
| One-method data set – fly round (n = 1,971) | Season | 1.0 |
|  | Sex | 1.6 |
|  | Wing length | 1.6 |
| One-method data set – trap (n = 224) | Season | 1.0 |
|  | Sex | 1.2 |
|  | Wing length | 1.3 |
| One-sex data set – females (n = 704) | Method | 1.5 |
|  | Season | 1.1 |
|  | Wing length | 1.4 |
|  | Ovarian category | 1.1 |
| One-sex data set – males (n = 1,491) | Method | 1.1 |
|  | Season | 1.0 |
|  | Wing length | 1.1 |
|  | Wing fray category | 1.0 |

**Additional file 1: Table S2**. Percentage predictor variable contribution of variance to the first three principal components (PCs) for different data sets.

| **Data set** | **Variables** | **PC1** | **PC2** | **PC3** |
| --- | --- | --- | --- | --- |
| 6.1 Whole data set (n = 2,195) | Method | 20.5 | 5.4 | 2.1 |
|  | Season | 3.2 | 28.3 | 96.0 |
|  | Sex | 30.8 | 8.1 | 0.2 |
|  | Wing length | 39.1 | 0.4 | 0.1 |
|  | Site | 6.4 | 57.8 | 1.6 |
| 6.2. Trap only data set (n = 224) | Season | 23.4 | 9.2 | 79.8 |
|  | Sex | 12.5 | 31.3 | 6.7 |
|  | Wing length | 33.2 | 0.1 | 2.4 |
|  | Site | 30.9 | 59.4 | 11.1 |
| 6.3. Fly round only data (n = 1,971) | Season | 1.2 | 46.2 | 91.9 |
|  | Sex | 45.8 | 1.2 | 2.1 |
|  | Wing length | 45.7 | 0.2 | 0.7 |
|  | Site | 7.3 | 52.5 | 5.2 |
| 6.4. Females only data set (n = 704) | Method | 21.0 | 16.9 | 6.1 |
|  | Season | 11.8 | 9.5 | 12.0 |
|  | Wing length | 37.8 | 0.6 | 2.0 |
|  | Site | 19.5 | 35.0 | 40.1 |
|  | Ovarian category | 10.0 | 38.0 | 39.8 |
| 6.5. Males only data set (n = 1,491) | Method | 25.9 | 3.0 | 11.0 |
|  | Season | 9.4 | 17.9 | 10.7 |
|  | Wing length | 33.1 | 5.8 | 0.2 |
|  | Site | 19.7 | 35.5 | 14.5 |
|  | Wing fray category | 11.9 | 37.8 | 63.5 |
| 6.6. Females only fly round data set (n = 558) | Season | 3.2 | 25.0 | 36.2 |
|  | Wing length | 34.3 | 0.3 | 0.9 |
|  | Site | 43.0 | 37.1 | 13.4 |
|  | Ovarian category | 19.5 | 37.5 | 49.4 |
| 6.7. Females only trap data set (n = 146) | Season | 20.9 | 30.7 | 20.8 |
|  | Wing length | 30.6 | 0.2 | 0.6 |
|  | Site | 33.4 | 18.8 | 28.7 |
|  | Ovarian category | 15.1 | 50.3 | 49.9 |
| 6.8. Males only fly round data set (n = 1,413) | Season | 14.7 | 14.1 | 38. |
|  | Wing length | 34.4 | 10.2 | 0.1 |
|  | Site | 34.4 | 39.2 | 18.5 |
|  | Ovarian category | 16.4 | 36.5 | 43.0 |
| 6.9. Males only trap data set (n = 78) | Season | 34.1 | 16.8 | 11.0 |
|  | Wing length | 20.8 | 2.7 | 17.1 |
|  | Site | 32.3 | 51.1 | 1.9 |
|  | Ovarian category | 12.8 | 29.4 | 70.1 |
